# Dietary intake, leisure time activities and obesity among adolescents in Western Sweden: a cross-sectional study

**DOI:** 10.1186/s12937-016-0160-2

**Published:** 2016-04-21

**Authors:** Anna Winkvist, Bodil Hultén, Jeong-Lim Kim, Ingegerd Johansson, Kjell Torén, Jonas Brisman, Heléne Bertéus Forslund

**Affiliations:** 1 Department of Internal Medicine and Clinical Nutrition, Sahlgrenska Academy, University of Gothenburg, Box 459, Gothenburg, SE-405 30 Sweden; 2 Occupational and Environmental Medicine, Sahlgrenska Academy, University of Gothenburg, Gothenburg, Sweden; 3 Cariology, Department of Odontology, Umeå University, Umeå, Sweden

**Keywords:** Diet, Obesity, Adolescents, Physical activity, Sweden, Cross-sectional study

## Abstract

**Background:**

Overweight and obesity among adolescents are increasing worldwide. Risk factors include dietary intake characteristics and high levels of physical inactivity. In Sweden, few large comprehensive population-based surveys of dietary intake and lifestyle among adolescents have been carried out. Thus, the purpose of the current study was to describe dietary intake and food choices as well as leisure time activities in relation to overweight and obesity in a total sample of all schoolchildren aged 15 years in Western Sweden.

**Methods:**

In 2008, a questionnaire was sent to all 21,651 adolescents born in 1992 in Västra Götaland Region, Sweden. Participation rate was 54.3 % (50.7 % girls/49.3 % boys). The questionnaire included a 73-item semi-quantitative food frequency questionnaire and questions on lifestyle. Results were evaluated against the Nordic Nutrition Recommendations and Swedish indicators of healthy diet and exercise habits. Associations with concurrent overweight and obesity were evaluated in multiple linear regression analysis.

**Results:**

Among girls, 49.5 % reached the goal of consuming fruit and vegetables at least daily, whereas for boys the figure was 34.4 %. Among both sexes, 15 % reached the goal of consuming fish at least twice weekly. Two-thirds of both sexes reached the goal of regular moderate or vigorous physical activity weekly. In total, 12.4 % were overweight and 2.4 % were obese. More girls than boys were underweight, whereas more boys than girls were overweight or obese (*p* < 0.001). Boys exhibited a more frequent intake of sodas and concentrated fruit juices, milk 3 % fat, bread and potatoes and fast food (*p* < 0.001). Frequent intake of candies and chocolate was reported by both sexes. Among girls and boys, living in rural areas, living in apartments and reporting no frequent leisure time physical activity were significant risk factors for being overweight or obese, also when adjusted for other risk factors.

**Conclusions:**

Dietary habits of adolescents in Western Sweden warrant improvements. Public health actions should be taken to increase consumption of fruit, vegetables and fish, and decrease consumption of sodas and candies and also to increase frequency of physical activity. These actions may be helpful in reducing risks for overweight and obesity.

## Background

Adolescents are becoming increasingly overweight and obese in rich as well as poor societies worldwide [1]. For example, in the US between the years 1980–2000 rates of obesity among adolescents 12–19 years old more than tripled [2]. Further, between the years 2003 and 2007 rates of obesity among adolescents 10–17 years old in the US increased by 6.1 % among boys and 17.6 % among girls [3]. In Europe, the annual increase in prevalence of childhood overweight was 0.1 % during the 1970s, 0.4 % during the 1980s, 0.8 % during the 1990s and 2.0 % by the 2000s [4]. These patterns are of clinical as well as public health concern, since obesity in childhood is associated with both short- and long-term health consequences [4–6].

Childhood overweight and obesity are the consequences of excessive energy intake relative to energy output together with a genetic disposition [6]. The mechanisms for such imbalance are complex though, involving both individual- and societal level factors. Individual factors include dietary intake characteristics e.g., frequency of eating, portion sizes, energy density, high fat intake and high intake of sodas and sugar. Dietary intake characteristics in childhood and adolescence are important to evaluate and act on if necessary, because they also predict dietary intake in adult life and thereby risk of chronic diseases [4, 7].

In Sweden, unfortunately not many large and comprehensive population-based surveys of dietary intake among adolescents have been carried out during the last decade. The National Food Agency in 2005, 2006 and 2008 carried out population-based telephone surveys of indicators of a healthy diet among Swedes aged 16–80 years. In 2008, in total only 211 persons in the age group 16–29 years were included [8]. Also, at three times (1989, 1997–1998 and 2010–2011), The National Food Agency has carried out more comprehensive dietary intake surveys among about 2000 Swedes; however the number of participants in the younger age groups are too small to be evaluated separately. Further, since 1985 the National Institute of Health in collaboration with the World Health Organization (WHO) carries out national lifestyle evaluations every four years. In 2009–2010, dietary intake and physical activity among school children 11–15 years old were evaluated as part of such a survey [9]. In addition, since 2004 the National Institute of Health yearly carries out a survey among 20,000 Swedes aged 16–84 years on lifestyle characteristics. However, this includes questions on frequency of intake of fruit and vegetables as sole indicators of dietary intake.

On the local level, diet information may also exist. For example, in the Gothenburg school district, school nurses have since 2004 systematically collected data on indicators of diet in addition to regular anthropometric measurements. Youth aged 11, 15 and 17 years answer questions on whether they daily consume breakfast, lunch and/or dinner as sole indicators of a healthy diet [10].

In short, there is a dearth of comprehensive information on a sufficiently large number of participants on dietary intake and food choices among adolescents in Sweden that go beyond a few indicators of a healthy diet. If the current obesity epidemic is to be reversed and future chronic diseases prevented, comprehensive dietary information is urgently needed. In the current study, the aim was to describe dietary intake and food choices as well as leisure time activities in relation to overweight and obesity in a total sample of all schoolchildren aged 15 years in Western Sweden.

## Subjects and Methods

### Study design

In 2008, a cross-sectional health survey among all adolescents living in Västra Götaland Region, born in 1992, was conducted. The Västra Götaland Region has 1.6 million inhabitants. The survey was initiated by the Committee of Public Health in Västra Götaland Region and conducted by the Institute of Medicine, University of Gothenburg. Focus of the survey was on prevalence of asthma and eczema in relation to environmental exposures [11]. As part of this, dietary intake and physical activity habits were also inquired about.

### Subjects

A questionnaire was sent by postal mail to 21,651 adolescents in Västra Götaland Region. Two reminders were sent out. In total, 11,753 responses were received (participation rate 54.3 % overall; 50.7 % girls and 49.3 % boys). Among adolescents born in Sweden or with Swedish parents, participation rate was 61 %, in comparison with 16 % among adolescents born abroad and 28 % among adolescents born in Sweden and with at least one parent born abroad. Further, adolescents with parents with lower levels of education had lower participation rates (38 % if mother had lower levels of education and 58 % if father had lower levels of education) than adolescents with parents with higher levels of education (58 % of mother had higher levels of education and 65 % of father had higher levels of education).

Among the 11,753 responders, 11,222 had complete information on dietary intake and anthropometry and these form the basis for the current analyses.

### Ethics statement

The study received full ethical approval by the regional ethics committee in Gothenburg, Sweden. All participating adolescents and their parents were informed about the study protocol by written information sent to the home. The adolescents thereafter signed informed consent sheets.

### Questionnaire

The ten-page questionnaire included pre-coded questions on place of upbringing, living area, type of residency, leisure time activities and screen time, ie time on computer for school work, for computer games, on internet and watching TV. The question on leisure time physical activity had five categories: rarely, sometimes light, regularly light, regularly moderate and regularly vigorous. In the analysis the categories were merged to three new categories; seldom or sometimes, light (rarely and sometimes light), regularly light (regularly light) and regularly, moderate or vigorous (regularly moderate and regularly vigorous). Self-reported weight (kg) and height (cm) were also asked about. Body mass index (BMI) was calculated as weight/height^2^. Further, categories of underweight, normal weight, overweight and obesity was identified as described for children and adolescents by Cole et al [12, 13] i. e. underweight BMI ≤ 17.69, ≤17.26, normal weight <24.17, <23.60, overweight <29.29, <28.60, obesity ≥29.29, ≥28.60 in girls and boys, respectively.

The questionnaire included a 73-item food frequency questionnaire (FFQ). This represents an adaptation of the 84-item FFQ used among adults in the Västerbotten Intervention Program since 1984 (see further details in [14]). In brief, the FFQ is semi-quantitative and portion sizes are reported on drawings of four plates of increasing portion size for the three categories staple food, meat/fish/vegetarian alternative and vegetables. Frequency of intake of the food items are reported on a nine-level scale, from “none” to “four or more times a day”. Daily intake has been calculated by multiplying frequency of intake by a portion size value either as indicated on the photographs, natural sizes such as an apple, or average portions sizes for the sex and age as described in a national survey [15, 16]. Energy and nutrient contents were calculated using the software MATSs (Rudans Lättdata, Västerås, Sweden) and the national food composition database [17]. The original 84-item FFQ has been validated with ten repeated 24-h recalls as well as with the biomarkers plasma beta-carotene, erythrocyte fatty acids and plasma B vitamins [16, 18, 19]. In this report, for some food items several frequency categories have been combined into larger categories.

### Statistical analysis

Socio-demographic characteristics of the adolescents were evaluated in relation to proportion meeting the indicators of healthy diet and exercise habits as defined by the Swedish National Food Agency [8], using Chi-square test. These indicators include consuming 500 g fruit and vegetables at least daily, consuming fish at least twice a week, and performing weekly regular moderate or vigorous leisure time physical activity. Unfortunately, in this study reported dietary intake did not encompass volumes but only frequencies; hence results on fruit and vegetable intake in relation to indicators are expressed as “any consumption at least daily”. Life style characteristics and dietary intake of macronutrients and food items were compared between girls and boys using Chi-square test for categorical variables and independent sample *t*-test for continuous variables.

Basal metabolic rate, BMR, was calculated based on sex, weight and height [20]. Total energy expenditure was calculated based on BMR and the average medium physical activity level (PAL) for that age (PAL = 1.73; [21]). Food intake level (FIL) was calculated as reported energy intake divided by BMR. The lowest and highest 5 % values of FIL were regarded as extreme and these individuals were excluded in the analysis of energy- and macronutrient intake, leaving 5013 girls and 4766 boys for these analysis.

Predictors of overweight and obesity were evaluated initially in bivariate regression analyses, using a selection of potential predictors including socio-demographic variables, indicators of a healthy diet and level of physical activity. Predictors that were found to be significant in these bivariate analyses were thereafter entered into multiple regression models. All regression models were run separately for girls and boys. Level of two-tailed significance was < 0.05 in all analyses. The statistical analyses were carried out using SAS, version 9.2, by SAS Institute Inc., Cary, NC, USA.

## Results

### Study population

The proportion of girls and boys in the dietary survey sample was similar (50.7 % vs. 49.3 %; Table 1). The majority of the adolescents grew up in Sweden, in urban areas and in a family that lived in a private residency. The proportion of adolescents reaching the Swedish indicators of a healthy dietary intake (ie consuming fruit and vegetables at least daily, and fish at least twice a week) and regular leisure time physical activity (ie regular moderate or vigorous physical activity), differed significantly in relation to the socio-demographic characteristics (Table 1). Girls were more likely than boys to reach the goal for fruit and vegetables (49.5 % vs. 34.4 %; *p* < 0.001), whereas slightly more boys than girls reached the leisure time physical activity goal (64.3 % vs. 60.3 %; *p* < 0.001). Among both sexes, 35 % never or seldom consumed any fish and only 15 % reported fish consumption ≥2/week. Reported intake of fish consisted of equal proportions of lean and fat fish (data not shown). Living in the rural area and staying in an apartment were significantly associated with less likelihood to reach the leisure time physical activity goal (*p* < 0.0001).

**Table 1:** Proportion of Swedish adolescents reaching the goals of a healthy dietary intake and regular physical activity in relation to socio-demographic characteristics (*n* = 11,222)

	Total sample	Fruit and vegetables at least daily	Fish ≥ 2 times per week	Regular physical activity weekly^a^
Sex (%)				
Girls	50.7	49.5**	14.8	60.3**
Boys	49.3	34.4	15.0	64.3
Grown up in Sweden (%)				
Yes	95.4	41.5***	14.7*	62.6**
No	1.3	52.1	21.2	47.8
Partly	3.3	54.6	18.5	58.7
Living area (%)				
Urban, inner city	15.2	45.8**	16.9*	61.3***
Urban, general	55.3	41.8	15.2	64.9
Rural	29.5	40.3	13.3	58.0
Housing style (%)				
Own residency	65.9	41.9	14.3	64.2***
Row house	14.8	42.2	16.2	66.3
Apartment	19.3	42.4	15.6	52.6

* *p* < 0.05; ** *p* < 0.001; and *** *p* < 0.0001, calculated by Chi-square test

^a^ Defined as regular moderate or vigorous physical activity

### Anthropometric and life style characteristics

In total, 12.4 % were categorized as overweight and 2.4 % as obese. Distribution of body mass index categories differed significantly between girls and boys (*p* < 0.0001; Table 2). More girls than boys were underweight, whereas more boys than girls were overweight or obese. Time spent on leisure time exercise, being outdoors and screen time also differed significantly between sexes (*p* < 0.0001), with boys spending more time on moderate or vigorous regular physical activity, being outdoors, and screen time. Here, the largest difference between sexes was seen for time spent on computer games. Having disturbed sleep or being sleepy during the day were significantly more pronounced in girls than in boys (*p* < 0.0001).

**Table 2:** Anthropometric and lifestyle characteristics of girls and boys in the Swedish adolescent health study (*n* = 11,222)

Characteristics	Girls	Boys	*p*-value
	*n* = 5,691	*n* = 5,531	
Body mass index (%)			
Underweight	9.5	5.0	
Normal	78.7	76.9	
Overweight	9.9	15.1	
Obese	1.9	3.0	<0.0001
Leisure exercise (%)			
Seldom or sometimes, light	20.6	21.3	
Regularly, light	20.1	15.5	
Regularly, moderate or vigorous	59.3	63.3	<0.0001
Time per wk spent outdoors (%)			
≤ 1 h	7.9	6.4	
1–7 h	50.5	43.6	
8–15 h	28.1	31.2	
> 15 h	13.5	18.8	<0.0001
Time per wk on computer for schoolwork (%)			
≤ 1 h	48.2	52.7	
1–7 h	46.0	42.0	
8–15 h	4.6	4.1	
> 15 h	1.2	1.2	<0.0001
Time per wk with computer games (%)			
≤ 1 h	75.7	18.2	
1–7 h	19.6	36.2	
8–15 h	3.3	24.2	
> 15 h	1.5	21.5	<0.0001
Time per wk on internet (%)			
≤ 1 h	13.2	16.5	
1–7 h	47.2	47.3	
8–15 h	27.6	23.0	
> 15 h	12.0	13.2	<0.0001
Time per wk watching TV (%)			
≤ 1 h	8.7	11.2	
1–7 h	50.8	51.3	
8–15 h	31.5	28.6	
> 15 h	9.1	8.9	<0.0001

* *p* < 0.05; ** *p* < 0.001; and *** *p* < 0.0001, calculated by Chi-square test

### Energy- and macronutrient intake

Total reported daily energy intake was significantly higher for boys than for girls (Table 3). Calculated total energy expenditure was (mean value + SD) 9.66 + 0.6 MJ/day for girls and 12.0 + 1.0 MJ/day for boys. Reported energy intake divided by calculated total energy expenditure was 80.7 % for girls and 87.5 % for boys, indicating some underreporting.

**Table 3:** Dietary intake of girls and boys in the Swedish adolescent health study (*n* = 9,779^a^)

Dietary component	Girls	Boys	*p*-value
	*n* = 5,013	*n* = 4,766	
Total energy intake (MJ/day)	7.8 ± 2.2^b^	10.5 ± 3.0	<0.0001
Total energy intake (kcal/day)	1867 ± 526	2516 ± 715	<0.0001
Protein (% of energy)	14.3 ± 2.6	15.2 ± 2.5	<0.0001
Carbohydrates (% of energy)	45.8 ± 6.7	43.3 ± 6.4	<0.0001
Fat, total (% of energy)	39.9 ± 7.3	41.4 ± 6.9	<0.0001
Fat, saturated (% of energy)	16.9 ± 4.1	18.3 ± 4.1	<0.0001
Fibre (g/day)	16.6 ± 7.6	18.2 ± 7.8	<0.0001
Fibre (g/MJ)	2.1 ± 0.7	1.7 ± 0.5	<0.0001
Sucrose (g/day)	33.8 ± 18.2	41.1 ± 23.6	<0.0001
Sucrose (% of energy)	7.3 ± 3.0	6.6 ± 3.1	<0.0001

^a^ Observations with extreme ratios between reported caloric intake and estimated basal metabolic rate (food intake level, FIL, were excluded i.e. FIL values below the 5^th^or above the 95th percentile value

^b^ Mean value ± SD, *T*-test was used

Dietary intake of macronutrients differed significantly between the sexes (*p* < 0.0001; Table 3). Girls reported a proportionally higher intake of carbohydrates, fibre and sucrose whereas boys reported a higher total energy intake as well as a proportionally higher intake of protein and total and saturated fat.

### Food choice

**Fig. 1:**
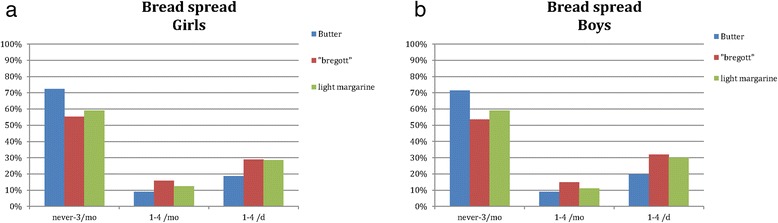
**a** and **b** Reported consumption of butter, butter-rapeseed oil blend (“Bregott”) and light margarine as bread spread among girls and boys (*p* < 0.05 girls vs. boys)

Intake of food items differed between girls and boys. Girls exhibited a significantly more frequent intake of fruit and vegetables than did boys (Table 4). Bread was only consumed daily by 70 % of boys and girls. Whole grain bread was consumed daily by 47 and 40 % girls and boys, respectively (*p* < 0.05). Daily consumption of butter as bread spread was reported among almost 20 % of both girls and boys (Fig. 1a and b), whereas daily consumption of the items “butter-rapeseed oil blend” and “low-fat margarine” each was reported among almost 30 % of girls and boys. Over 90 % of the girls and boys consumed ordinary margarine never or ≤ 3 times/month. Only a small proportion of girls and boys reported a daily intake of cheese (Table 4), most of which was high-fat cheese (data not shown). Among staple foods, potatoes were the most commonly reported item for both sexes, with around 30 % reporting consuming potatoes once a day or more often (girls 24 % vs. boys 32 %, *p* < 0.05). This staple food was followed by pasta, rice and lastly bulgur or couscous.

**Table 4:** Frequency of intake of commonly consumed food items among Swedish girls and boys (*n* = 11,222)

Food item	Frequeny of intake					
	<1/week	≥1/week	≥2/week	1/day	≥2/day	*p*-value^a^
Fruit (%)						
Girls	1.5	10.5	27.3	38.1	22.7	
Boys	4.3	16.0	30.8	32.5	16.3	<0.0001
Vegetables (%)						
Girls	1.4	6.1	23.3	49.3	19.8	
Boys	3.5	10.2	31.9	41.0	13.4	<0.0001
Bread (%)						
Girls	1.4	3.8	25.2	48.0	21.6	
Boys	1.8	4.2	23.7	41.8	28.5	<0.0001
Cheese						
Girls	44.1	11.9	29.2	10.0	4.5	
Boys	38.1	11.7	30.5	11.7	8.0	<0.0001
Potatoes (%)						
Girls	0.5	8.8	66.6	21.9	2.2	
Boys	0.4	6.1	61.2	28.8	3.4	<0.0001
Pasta (%)						
Girls	8.2	27.2	62.5	1.5	0.6	
Boys	7.2	26.7	62.8	2.3	1.0	0.0013
Rice (%)						
Girls	22.4	40.4	35.0	1.5	0.7	
Boys	20.3	39.8	37.1	1.6	1.2	0.0014
Bulgur, couscous (%)						
Girls	86.5	8.3	4.8	0.3	0.04	
Boys	90.0	6.1	3.2	0.4	0.3	<0.0001

^a^Chi-square test was used

**Fig. 2:**
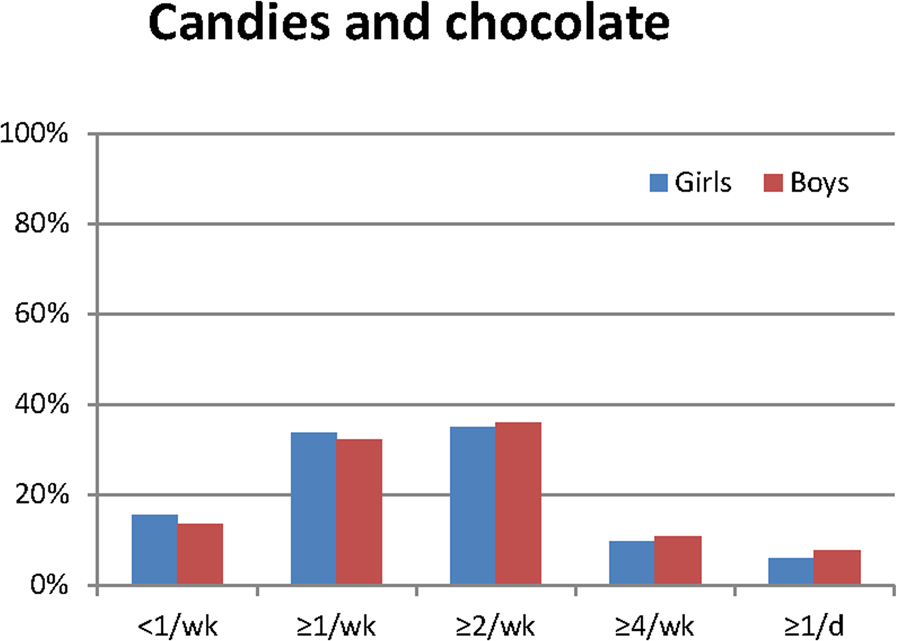
Reported consumption of candies and chocolate among girls and boys (*p* < 0.05 girls vs. boys)

**Fig. 3:**
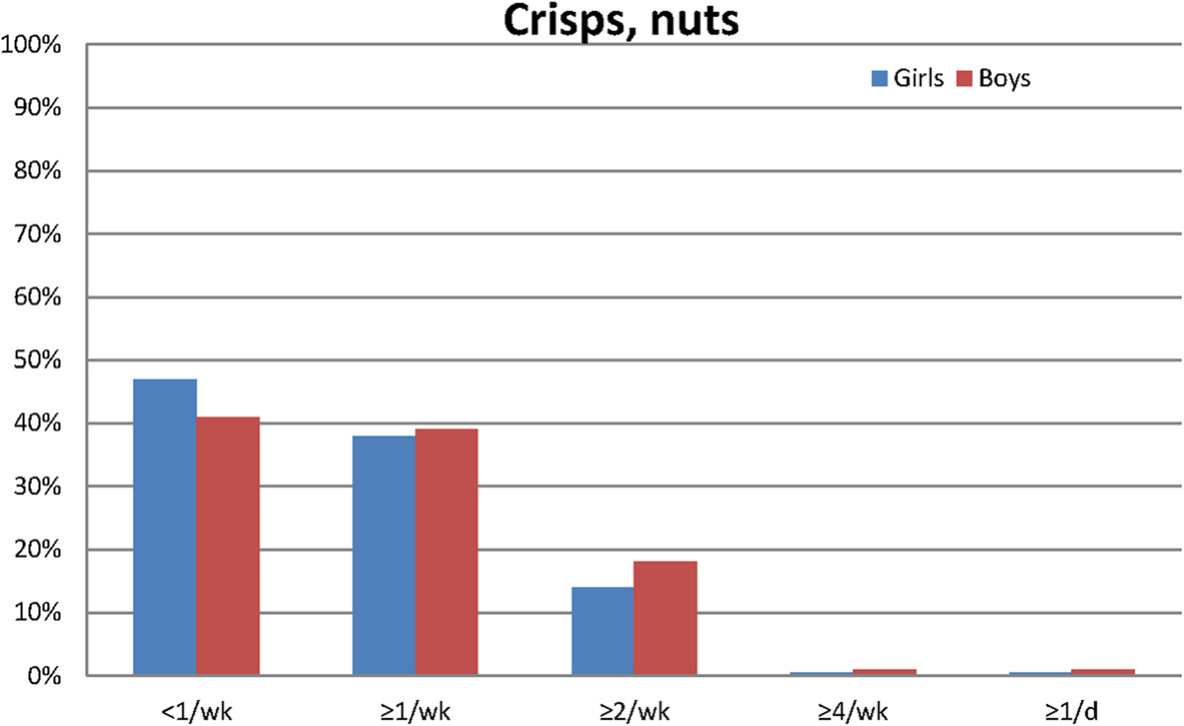
Reported consumption of crisps and nuts among girls and boys (*p* < 0.001 girls vs. boys).

Most commonly consumed type of milk was the 1.5 % fat type (Table 5). Boys reported a more frequent consumption of the 3 % fat type milk than did girls. Reported intake of soda light products was low among both girls and boys. Overall, boys reported a significantly more frequent intake of sodas and concentrated fruit juices (also when excluding low energy sodas; data not shown). Daily consumption of sodas and concentrated fruit juices was higher among boys (20 %) than among girls (10 %; *p* < 0.05). Boys reported significantly more frequent consumption of fast food (hamburgers, French fries, sausages, hot dog, pizza) than did girls (Table 6). Candies and chocolate were reported to be consumed four times a week or more by 16 % of the girls and 18 % of the boys (Fig. 2, *p* < 0.05 girls vs. boys), whereas crisps and nuts were reported to be consumed less than once a week by 47 % of girls and 41 % of boys (Fig. 3, *p* < 0.0001).

**Table 5:** Frequency of intake of beverages among Swedish girls and boys (*n* = 11,222)

Food item	Frequency of intake					
	<1/week	≥1/week	≥2/week	1/day	≥2/day	*p*-value^a^
Milk, 3 % fat (%)						
Girls	78.8	4.0	6.7	4.4	6.2	
Boys	69.8	4.7	8.7	5.7	11.1	<0.0001
Milk, 1.5 % fat (%)						
Girls	32.9	5.8	19.5	16.0	25.8	
Boys	29.0	4.7	16.9	14.7	34.7	<0.0001
Milk, 0.5 % fat (%)						
Girls	67.7	4.2	9.5	7.6	11.0	
Boys	72.7	3.9	6.9	5.2	11.2	<0.0001
Sodas and concentrated fruit juices (%)						
Girls	18.8	41.4	29.5	7.1	3.1	
Boys	7.3	31.9	41.0	13.7	6.1	<0.0001
Juice (%)						
Girls	30.7	17.2	31.1	15.1	5.9	
Boys	29.2	18.0	30.8	15.3	6.7	0.179

^a^Chi-square test was used

**Table 6:** Frequency of intake of fast food items among Swedish girls and boys (*n* = 11,222)

Food item	Frequency of intake					
	<1/month	≥1–3/month	≥1/week	≥2–3/week	≥4/week	*p*-value^a^
Hamburgers (%)						
Girls	28.5	59.7	9.3	1.7	0.8	
Boys	15.9	60.5	18.0	3.7	1.9	<0.0001
French fries (%)						
Girls	25.9	56.1	12.6	3.7	1.7	
Boys	18.8	54.3	18.3	5.8	2.8	<0.0001
Sausages, hot dog (%)						
Girls	25.8	40.1	26.6	6.7	0.9	
Boys	14.4	38.9	37.3	7.5	1.9	<0.0001
Pizza (%)						
Girls	27.6	63.8	6.8	1.3	0.5	
Boys	14.8	62.3	16.7	4.1	2.0	<0.0001
Fast food (%)^b^						
Girls	1.8	4.8	57.6	30.4	5.5	<0.0001
Boys	0.3	2.0	43.5	42.3	11.9	

^a^Chi-square test was used ^b^ Fast food = hamburgers + French fries + sausages/hot dog + pizza

### Risk factors for overweight or obesity

In bivariate regression analyses, boys exhibited a significantly increased risk of being overweight or obese compared to girls (OR 1.65, 95%CI 1.48, 1.83; Table 7). Low reported frequency of intake of vegetables, but not of fruit, was significantly associated with a 20 % increased risk of being overweight or obese (OR 1.20, 95%CI 1.08, 1.33). Low consumption of fish was associated with a 17 % increased risk of being overweight or obese (OR 1.17, 95%CI 1.04, 1.32). Further, irregular physical activity, less time spent outdoors, more time spent on computer games were significantly associated with increased risk of being overweight or obese.

**Table 7:** Risk factors for being overweight or obese among Swedish girls and boys (*n* = 11,222)

Risk factor	Bivariate model	Multivariate models^a^	
	Odds Ratios (95 % CI)	Odds Ratios (95 % CI)	
Sex			
Girls	1.0		
Boys	1.65 (1.48, 1.83)		
		Girls	Boys
Grown up in Sweden			
Yes	1.0		
No	0.85 (0.52, 1.38)		
Partly	0.89 (0.66, 1.21)		
Living area			
Rural	1.0	1.0	1.0
Urban, general	0.79 (0.71, 0.89)	0.74 (0.61, 0.91)	0.82 (0.70, 0.98)
Urban, inner city	0.70 (0.59, 0.83)	0.55 (0.40, 0.75)	0.64 (0.49, 0.84)
Housing style			
Apartment	1.0	1.0	1.0
Row house	0.75 (0.62, 0.90)	0.67 (0.49, 0.92)	0.71 (0.55, 0.93)
Own residency	0.87 (0.76, 0.99)	0.74 (0.58, 0.94)	0.77 (0.63, 0.95)
Vegetables daily			
Yes	1.0	1.0	1.0
No	1.20 (1.08, 1.33)	1.01 (0.84, 1.22)	1.05 (0.91, 1.22)
Fruit daily			
Yes	1.0		
No	0.98 (0.88, 1.08)		
Fish weekly			
Yes	1.0	1.0	1.0
No	1.17 (1.04, 1.32)	1.03 (0.84, 1.26)	1.09 (0.91, 1.30)
Regular physical activity			
Yes	1.0	1.0	1.0
No	1.66 (1.49, 1.84)	1.57 (1.31, 1.87)	1.56 (1.34, 1.81)
Time spent outdoors per wk			
> 7 h	1.0		
≤ 7 h	1.12 (1.01, 1.24)	1.14 (0.95, 1.36)	1.04 (0.90, 1.21)
Time spent on computer games per wk			
> 7 h	1.0	1.0	1.0
≤ 7 h	0.71 (0.64, 0.80)	0.81 (0.55, 1.19)	0.99 (0.86, 1.15)
Time spent on internet per wk			
> 7 h	1.0		
≤ 7 h	0.98 (0.88, 1.10)		
Time spend watching TV per wk			
> 7 h	1.0		
≤ 7 h	0.90 (0.81, 1.00)		

^a^All variables that were significant in the bivariate models were included in the multivariate models for girls and boys, respectively

The multivariate regression models included all variables that were significant in the bivariate analyses. To also capture whether sex modified the effect of other risk factors on the risk of being overweight or obese, these models were run separately for girls and boys. Here, among both girls and boys, living in urban areas and living in row house or own residency were signficantly associated with less risk of being overweight or obese, compared to living in rural areas or living in apartments. Also for both sexes, reported infrequent physical activity was significantly associated with an almost 60 % increased risk of being overweight or obese in multivariate analyses. Daily intake of vegetables or fruit and weekly intake of fish was not associated with the risk of being overweight or obese among the girls or boys.

## Discussion

A comprehensive survey of dietary intake and lifestyle factors was carried out among the total population of adolescents in Västra Götaland Region, Sweden (*n* = 21 651 in 2008). Important findings included that more girls than boys reached the goal of consuming fruit and vegetables at least daily (49.5 % vs. 34.4 %), that only 15 % of both sexes reached the goal of consuming fish at least twice weekly and that about two-thirds of both sexes reached the goal of regular moderate or vigorous leisure time physical activity weekly. In total, 12.4 % of all adolescents were overweight and 2.4 % were obese; both being more prevalent among boys than girls. Girls reported a proportionally higher intake of carbohydrates, fibre and sucrose, whereas boys reported a proportionally higher intake of protein as well as total and saturated fat. Boys reported more frequent consumption of sodas and concentrated fruit juices, milk with 3 % fat, bread, potatoes and fast food. Significant risk factors for being overweight or obese included living in rural areas, living in apartments and reporting no frequent leisure time physical activity.

We have chosen to compare our results with indicators of nutrient quality (ie, intake of fruit and vegetables and fish) developed by the National Food Agency [8] as well as with the Nordic Nutrition Recommendations on consumption of fat, saturated fat, sugar and fibre [21]. In addition, we have highlighted the consumption of butter and margarine because of trends in fat consumption recently noted among Swedish adults [22]. Based on population-based cross-sectional consumption data collected with the same methodology during 1986–2010, this study described a significant decrease in fat consumption between 1986 and 1992 and a significant increase from 2002 for women and from 2004 for men. During this period, among both sexes use of butter as bread spread remained constant, use of low fat margarine decreased and use of butter-rapeseed oil blend increased. Concurrently, serum cholesterol levels decreased during 1986–2004 and increased after 2007. Hence, it is of interest to investigate recent patterns of fat intake also among Swedish adolescents.

The study is unique in that it invited the total population of 21, 651 adolescents in Västra Götaland Region. The participation rate was 54.3 %, which may be compared with the Swedish national dietary survey Riksmaten, with a participation rate of 36 % [23]. Thus, the participation rate in our survey was in the upper range of what could be expected. Still, due to the varying participation rates among subgroups of adolescents, our results are likely not representative for foreign-born adolescents or adolescents with parents of short education.

Research has shown that by the age of 8–10 years, children can report their food intake as reliably as their parents and by adolescence cognitive abilities are fully developed, although issues of motivation and body image may hinder adequate reporting [24]. Thus, recall errors are to be expected and include both underreporting and overreporting. Previous research on adolescents in Göteborg, Sweden, found that over-reporting was more common among boys and underreporting was more common among girls [25]. Also among adolescents, underreporting tends to increase with increasing overall eating frequency as well as with higher body mass index, and less common foods are more easily forgotten than are main courses [24, 26]. Still, modified dietary history and food frequency questionnaire are likely able to rank adolescents according to most nutrients [25, 27], and adolescents do have the capacity to use photographs to estimate portion sizes [28].

In our survey, energy intake was underreported as indicated by differences between reported energy intake and calculated total energy expenditure; a difference similar to that reported by others for this age group [26]. In that study, underreporting of energy intake among adolescents from seven days food records, in comparison with total energy expenditure measured by the doubly labeled water method, was 78.3 % for girls and 81.9 % for boys. In our survey, the magnitude of underreporting for other nutrients is probably similar to that of energy. In our evaluations of macronutrient intake, all values are expressed as percentage of energy. Hence, effects of overall underreporting are less likely to bias comparisons of macronutrient intakes, although possible bias due to selective underreporting remains unknown. Still, diet intake data based on food frequency questionnaires with a limited number of food items are not expected to cover the total daily intake. Hence, such data should not be used in comparisons with absolute recommendations but are suitable for comparisons between subgroups, which is how data are used in this paper.

The sex-specific consumption patterns observed in our study are supported by previous Swedish findings. A national survey in 2009–2010 among 11, 13, and 15 year old school children noted that girls reported a higher consumption of fruit and vegetables whereas boys reported a higher consumption of sodas and candies and also more time on physical activity and computers [9]. These patterns were established already among the 11-year-olds and differences increased with age. Such patterns also have been reported from other Nordic countries [29]. Also, the clustering of unhealthy consumption patterns in this age group has been reported also in other Nordic countries, e.g. Norway [30, 31].

Results on reported consumption of healthy and unhealthy food items among Swedish adolescents vary. In the national survey in 2009–2010 [9], among the 15-year-olds only 27 % of the girls and 22 % of the boys reported consuming fruits daily or more often and only 39 % of the girls and 31 % of the boys reported consuming vegetables daily or more often. Furthermore, a survey from Gothenburg, Sweden, among 18-year-olds [32] reported a daily intake of fruit and berries among 40 % of the girls and 21 % of the boys, and a daily intake of vegetables among 59 % of the girls and 36 % of the boys. Our recent survey thus found higher proportions of both girls (61 %) and boys (39 %) reporting at least daily consumption of fruits, and higher proportions of both girls (69 %) and boys (54 %) reporting at least daily consumption of vegetables. In our survey, sodas and concentrated fruit juices consumed at least daily were reported among 10 % of the girls and 20 % of the boys, which is slightly higher than previously reported in a survey from Gothenburg, Sweden [33] (girls 3 %, boys 8 %) and in the national survey in 2009–2010, (girls 6 %, boys 11 %) [9]. International comparison reveals that the proportion reporting daily fruit consumption in Sweden was below the international average for both sexes for all 40 countries included in the WHO international comparison [9].

Possible explanations for the more healthy consumption patterns reported in our survey include the lower representation of foreign-born adolescents and adolescents with parents of short education. Also, the proportion of adolescents living in apartments in our survey (19.3 %) was lower than the corresponding national average for 0–24 years living in apartments (33 %) [34]. Finally, it is possible that our survey captures a trend over time of improved dietary intake in this age group. Previous data on secular trends of vegetables and fruit consumption among adolescents in Sweden is sparse. According to the annual surveys from Public Health Agency of Sweden the consumption of fruit and vegetables 1–5 times/day has increased the last ten years among boys 16–29 years of age, although the increase is negligible among girls [35].

Among girls, we found lower prevalence of both overweight (9.9 % vs. 15.1 % among boys) and obesity (1.9 % vs. 3.0 % among boys). Similarly, the national 2009/10 survey found a prevalence of self-reported overweight and obesity of 20 % among boys and 8 % among girls [9]. The survey was part of an international initiative and presented an average prevalence of overweight and obesity among 15-year olds in 40 countries of 21 % among boys and 12 % among girls [36]. Further, prevalence of measured overweight and obesity among 16-year-olds in a small national school based sample from 2001 were almost identical with that of ours for boys but somewhat higher for girls [37]. Likewise, more recent measured data on adolescents from southeastern Sweden reported similar prevalence of overweight and obesity for boys but again somewhat higher for girls than our survey [38]. In contrast, in an environment of high education in Uppsala, Sweden, the prevalence of measured overweight and obesity was much lower for 16-year old boys but similar to our prevalence for girls [39]. In sum, the higher prevalence of overweight and obesity among boys found in our study are consistent with that of many other reports and warrant attention.

In our survey, significant risk factors for being overweight and obese included living in rural areas, living in apartments and reporting no frequent leisure time physical activity. Surprisingly, infrequent intake of fruits, vegetables and fish were not significant risk factors in the multivariate models. Only these indicators of a healthy diet were included in these analyses; hence it is possible that other components of the diet may have relation with risk of being overweight and obese.

The Norwegian national survey identified social class, time spent watching TV or in front of a computer and breakfast frequency as risk factors for being overweight among 13 -year-olds [27]. Further, more frequent TV viewing in adolescence and early adulthood was in the 1958 British birth cohort associated with greater BMI gains through to mid-adulthood and with central adiposity in mid-life [40]. In Norway, among young teenagers a twenty-month change in screen time was positively associated with changes in the consumption of sodas and unhealthy snacks in the same period [41]. Clustering of extended screen time and high consumption of sodas as well as infrequent breakfast habits has been reported among 11–12 year olds in Gothenburg, Sweden [42]. In our survey, extended screen time was in bivariate analyses significantly associated with increased risk of being overweight or obese. However, the significance disappeared in multivariate analyses, perhaps due to collinearity with factors such as time spent outdoors and regular physical activity. We noted a sex difference with boys reporting more screen time than girls, especially for time on computer games. In Sweden, total screen time has increased markedly for 15-year-olds since 2001 even though time for TV/DVD takes a smaller part [9]. From a health perspective, this is a reason for concern.

In summary, our results on reported intake of healthy and unhealthy food items among adolescents in western Sweden describe sex differences similar to those of previous Swedish studies, with girls reporting more healthy habits than boys. Overall, our results point to a somewhat more healthy consumption pattern than found in previous studies, although this may partly reflect misreporting and selection bias. Prevalence of overweight and obesity support previous findings, and risk factors for being overweight and obese include socio-demographic characteristics such as living in rural area and living in apartments, and having low reported frequency of leisure time physical activity. The latter likely also reflects more frequent screen time, a phenomenon that has increased in this age group in Sweden in the last decade. In a public health perspective, these findings are a reason for concern. Public health actions should be taken to increase physical activity and consumption of vegetables, fruit and fish, as well as decrease consumption of sodas and candies with special attention to sex differences.

## Abbreviations

BMI: body mass index
BMR: basal metabolic rate
CI: confidence interval
FFQ: food frequency questionnaire
FIL: food intake level
OR: odds ratio
PAL: physical activity level
WHO: World Health Organization
